# A proposed protocol for the registration of evidence-based Educational Psychology programs

**DOI:** 10.3389/fpsyg.2022.954475

**Published:** 2022-11-07

**Authors:** Jesús de la Fuente, Manuel Mariano Vera-Martínez, Francisco Javier Peralta-Sánchez, José Manuel Martínez-Vicente

**Affiliations:** ^1^School of Education and Psychology, University of Navarra, Pamplona, Spain; ^2^School of Psychology, University of Almería, Almería, Spain; ^3^School of Psychology, University of Granada, Granada, Spain; ^4^General Council for Psychology, Madrid, Spain

**Keywords:** evidence-based intervention, protocol for evaluating Educational Psychology intervention programs, national database, professional practice, general psychological society

## Abstract

The turn to evidence-based interventions is significant for Psychology in general and for Educational Psychology in particular. Although there is a great deal of published evidence for program validation, there is currently no protocol for the evaluation of educational psychology evidence-based intervention programs and there is no General Register of such programs to act as a central information depository. This article has four objectives: (1) To assess the significance of the turn to *Evidence-Based Programs* in the context of today’s Knowledge Society and Research and Development, Transfer and Innovation (R&Di) activities; (2) To provide a *Classification of Programs* based on the degree of specialization required for appropriate professional use in line with the requirements of the Code of Ethics in Psychology; (3) To tentatively propose a *Protocol for the Registration of Evidence-Based Educational Psychology Programs*; and (4) Finally, to identify some implications of the protocol. We conclude that there is a need for a *General Register* of validated programs.

## Introduction

The development of Psychology as a science and of the discipline of Educational Psychology has led to a proliferation of conceptual models in each area of study and the development of assessment and intervention tools to implement those models in professional practice. A Google Scholar search for *Psychological Intervention Programs* gave some 360,000 results, *Educational Intervention Programs* produced some 4.5 million publications, and *Educational Psychology Intervention Programs* gave 3 million results. Those figures demonstrate the large volume of work concerning the creation and use of Programs, properly so called.

The term *Intervention Program* describes a set of components or structured decisions and actions intended to have a particular effect in a given personal or social environment (Berdychevsky et al., 2022; Gijzen et al., 2022). A material proportion of published Psychological Programs have been created by members of the profession and university academics; publishing houses and university knowledge transfer technology companies have generally undertaken publication and sale. The trend is if anything accelerating with, for example, the current promotion by Spain’s University System of new processes, products, and services by way of transfer to the wider community and the profession through the Spanish knowledge transfer program, *Six Years of Transfer,* 2012*–2018* (Repiso et al., 2019). The European Union has also attributed considerable significance to technology transfer.

However, despite those developments and despite copious attempts to provide a basis for program validation (Sánchez-Meca et al., 2002), there is as yet no general protocol for the assessment of scientific quality or underlying scientific evidence so as to determine whether to endorse a Psychological Intervention Program. However, although much attention has been devoted to the evaluation, validation, and classification of psychological assessment tools by researchers (Moreno et al., 2018; Muñiz and Fonseca-Pedrero, 2019) and practitioners (Commission for Tests Edited in Spain, 2012), the same is not true of *Psychological Intervention Programs* in use in professional practice.

Consequently, this article has four objectives: (1) To assess the significance of the turn to *Evidence-Based Programs* in the context of today’s Knowledge Society and Research and Development, Transfer and Innovation (R&Di) activities; (2) To provide a *Classification of Programs* based on the degree of specialization required for appropriate professional use consistent with the requirements of the Code of Ethics in Psychology; (3) To tentatively propose a *Protocol for the Registration* of *Evidence-Based Educational Psychology Programs* for Educational Psychology Intervention Programs; and (4) Finally, to identify some implications of the protocol. We conclude that there is a need for a *General Register* of validated programs.

## The turn to evidence-based programs in the context of the knowledge society of the 21st century

### Science in the knowledge society

*The Research*, *Development*, *and Innovation (R&Di)* chain as it operates in the Knowledge Society has achieved much, as we can see by considering the links in that chain individually (de la Fuente and Vera-Martínez, 2010; de la Fuente et al., 2012; Martínez-Vicente et al., 2020):

1. Research and the accumulation of scientific knowledge have provided progressively more evidence and led to the development of more accurate scientific models that have been generative of more scientific and more reliable predictions. For example, in Educational Psychology, there is a large body of scientific evidence in specific areas and conceptual models based on that evidence in each of those areas (Harris et al., 2012; de la Fuente and Justicia, 2018).
2. That has in turn led to technological development in the form of innovative assessment and intervention techniques based on that growing scientific knowledge. Strategies and techniques for assessment (Tests) and intervention (Programs) fall into this category: they are technological (in the true sense of the term) developments in a conceptual domain in a given area of knowledge and as such should be developed and applied scientifically. Consequently, the knowledge they are based on—the underlying theoretical model and other principles and assumptions—should be clear.
3. The transfer of such innovations for use in a given population requires the systematic collection and assessment of evidence to identify the results of an intervention. This closes the circle by allowing theoretical prediction and outcomes to be compared and contrasted.
4. University *Knowledge Transfer Departments* are the channel through which those activities are carried out. In essence, the mission of Knowledge Transfer Departments is to encourage research, development and (ultimately) knowledge transfer. They also promote the appropriate use of programs based on robust evidence that allows the effects of interventions to be predicted with a high level of confidence.

For all those reasons, the *Turn to Evidence-Based Programs* has become more prevalent.

### The turn to evidence-based programs

The *Turn to Evidence-Based Program* has developed with the notion of scientific evidence in other areas (Hemsley-Brown and Sharp, 2003; Chaves and Ramírez, 2020; See, Walter et al., 2005; See et al., 2019) and in Psychology (de la Fuente et al., 2019b). It is a consequence of scientific maturity and adoption of the principle that psychological interventions developed in the R&Di value chain should be evidence-based (de la Fuente and Vera-Martínez, 2010, 2021; de la Fuente et al., 2019a).

In education, two recent seminal articles by Slavin (2017, 2019) lay out the progress that would be achieved by the level of evidence behind each intervention program being available to the educational system. He posits that such information would lead to: (1) well-founded decision-making by professionals; (2) routine accreditation of evidence of the effects of such programs as they are disseminated; and (3) the adoption of systematic evaluation of programs in the field of education. We would add: (4) a statutory obligation on creators and businesses that market programs to provide validation information; (5) scientific validation of processes of intervention and consolidation of businesses and knowledge transfer departments in educational organizations of whatever kind, led by people who are qualified to produce empirical evidence and to test such processes (de la Fuente and Zapata, 2012).

The provision of evidence for any treatment or intervention program is a key to moving toward high-quality scientific psychological interventions. In many fields of Health Sciences (Villalbí, 2001) and in educational psychology (de la Fuente, 2020) it is increasingly accepted that interventions should be evidence-based. (For an explanatory video, see be evidence-based (For an explanatory video, see (Spanish): https://www.youtube.com/watch?v=M70bPL63nvk). The use of evidence-based interventions requires the adoption of criteria for the evaluation and review of intervention programs.

Slavin (2002, 2008, 2013, 2017, 2019) has promoted a national searchable database in the United States (Slavin, 2002, 2019) to contain the evidence and features of each intervention program. Such a database would provide significant support to decision-making in the selection of intervention programs. However, it raises a further, as-yet unresolved problem in relation to a Protocol of Evaluation Criteria for Evidence-Based Educational Psychology Programs (de la Fuente, 2020). That issue is explained below.

## Types of programs: Educational, Psychopedagogical/Pedagogical, and Educational Psychology counseling

Although it may seem obvious, words are currently being used indiscriminately in a way that makes it hard to identify the area of study from which Educational Psychology Intervention Programs originate. Because of that lack of clarity, three types of intervention program are being used without their natures and scopes being fully clear.

For these purposes, an Intervention Program can be characterized as a suite of formal decisions, i.e., planned, explicit decisions that are contextualized, systematized, and intended to have specific effects. Those effects may be intended to arise within a family, a school, among teachers or students and concern the processes of development, learning, or teaching in any of those settings that in the aggregate constitute the process of education. We can categorize intervention programs as follows. Table 1 is a tabulation of this classification.

**Table 1:** Categorization of intervention program.

**Program level**		**Concept**	**Domain**	**Focus**	**Delivered by**
1.	Educational	Educational	School	Generalist	Subject teacher
2.	Pedagogical	Psychoped	Psychopedagogy	Specific	Pedagogical or Guidance Counselor
3.	Educational Psych	Educational Psych	Educational Psych	Specific	Educational Psychologist

### Educational intervention programs

Educational Intervention Programs fall conceptually and functionally under the umbrella of Educational Science, which is the suite of disciplines dedicated to the scientific study of different aspect of education in specific societies and cultures. That group of disciplines reflects the integration of all the sciences that may focus on a given educational problem, namely: Pedagogy, Comparative Pedagogy, General and Specific Didactics, Sociology, Anthropology, Philosophy, Economics, Theology (where applicable), and Psychology. Consequently, we can use the term Educational Intervention Program for any program that: (1) addresses an educational problem from an educational perspective; (2) falls within the combined field of any of those disciplines; and (3) is capable of being implemented by a professional educator with no specific qualifications beyond a teaching qualification and subject expertise in the curricular subject to which the Program relates. Most subject-specific educational programs come under this heading. Educational intervention programs are generally used by teachers in schools.

### Pedagogical intervention programs

This category is made up of professional programs that are closer in concept to interventions as understood in Psychopedagogical (a classification used in Spain), Pedagogical, and Educational Guidance (Aubrey, 1988; Bisquerra et al. 1998). Although, the term “psychopedagogical” arguably merits an article of its own—not this one—it can be taken to mean three things: (1) the program addresses an educational problem from a psychopedagogical perspective, i.e., it includes elements from psychology; (2) the program is a professional guidance program; and (3) such programs tend to have a generalist educational science focus but with some specialist elements from pedagogy or psychology, without necessarily having a specifically educational psychology focus. This category includes many guidance-focused Intervention Programs created, proposed and adopted by Ministries of Education for use in school guidance programs delivered by secondary school teachers. It is school guidance counselors in particular who generally use pedagogical intervention programs.

It should be noted that Psychopedagogy in a sense makes Psychology and the other disciplines subordinate to Pedagogy and Didactics, its core disciplines. These programs are essentially pedagogical in focus, with some ancillary elements or insights from other disciplines, such as Psychology. Thus, such programs are not necessarily based on models, assessment techniques, or interventions from within Psychology. This can be exemplified by comparing a Health Education Program drawn up as part of the general education of students (Ministry of Health of La Rioja, 2019) and a program that is expressly from within Educational Psychology. The Ministry of Education (Spain) has created a website related to Evidence-Based Education (Ministry of Education, 2020).

### Educational Psychology intervention programs

This category is for programs whose conceptual foundations come from Developmental and Educational Psychology, such as the Psychology of Education and related psychological disciplines, namely: Developmental Psychology, Psychology of Learning Difficulties, and the Psychology of Vocational Development. These Programs are generally more specialist. They are specifically based on educational psychology theoretical models and on scientific evidence from each of the disciplines mentioned, such as evidence from *Educational Psychology* (de la Fuente and Justicia, 2018) and other specialist work in the field, whether in Europe or from the APA in the United States (Working Group IAP/APA, 2013). Their application requires: (1) specific educational psychology training or degree; (2) specialist knowledge of the explanatory models and the scientific evidence in the area; and (3) specific training in a particular Intervention Program. The requirements of the Code of Ethics of Psychology for qualifications in Psychology for those who implement psychological tests and techniques and programs for psychological intervention are of particular significance here (Consejo Oficial de la Psicología de España, 2008). Article 19 of the Code reads as follows: “Any Psychology materials, whether related to assessment and intervention or to treatment, may only be used by Psychologists. Psychologists may not provide such materials to unqualified people. Psychologists will manage or, as the case may be, ensure proper custody of documents pertaining to Psychology.” Consequently, these particular programs should be used by Educational Psychologists.

## Essential features of a proposed protocol for the registration of evidence-based Educational Psychology programs

The protocol for the registration of evidence-based programs (de la Fuente, 2020) contains the following sections.

### Professional context

#### Professional context

This first level of categorization is the general area of a Program. Whether a program is educational, psychopedagogical or from educational psychology is a threshold issue because the protocol is, as explained, intended for educational psychology programs.

#### Intervention area of the program

Beyond that basic information, the area in which a program makes an intervention must be clearly described. In the case of the educational psychology programs for which the Protocol is intended, we need to know whether a given Intervention Program has indeed been designed for an educational psychology program.

##### Intervention programs to optimize processes of development, teaching, and learning

Interventions in the processes of human development, in programs focused on any aspect of human development (physical, psychomotor, personal or emotional, social-ethical, cognitive, and linguistic). If a Program concerns learning, it will seek to improve the learning process and the skill of learning to learn in any of its conceptual, procedural or attitudinal aspects. Programs to improve teaching also fall into this category.

##### Intervention programs to address difficulties in the processes of development and learning

This group is for all Intervention Programs specific to the adjustment, treatment and improvement of any difficulties and problems that arise in the macroprocesses of development and learning.

##### Academic and vocational guidance programs

Specific academic guidance and career-orientation programs are in this group.

##### Programs of educational psychology research, technology development, and innovation (R&Di)

This category includes all Intervention Programs associated with computer-supported innovation relating to the improvement of a process, product, or service within educational psychology: Apps, Computer Programs, etc.

#### Specific intervention area of the program

Level 3 is for the specific type of treatment within the broader Level 2 area. For example, an Emotional Intelligence program would be in the specific area of *Optimization of Personal and Social Development*. If a program is intended to support learning under stress conditions, it would be in the specific area of *Optimization of the Learning Process*.

#### Type of program

It is important to capture the nature of the Program in terms of the level at which intervention is made.

##### Preventive (primary)

Programs intended to optimize the processes of development or learning in their initial phases before the emergence of educational problems.

##### Palliative (secondary)

Programs aimed a rechanneling development processes when the first symptoms of maladjustment or difficulty have appeared.

##### Treatment (tertiary)

Programs aimed at intervening when problems have manifested.

##### Retrospective (stage 4)

Programs aimed at prevention of the unwanted side effects of prior interventions.

### Program details

This section contains:

1. The basic details of a program: its name, the specific problem addressed, year of creation, the authors, and any registered intellectual property rights (IPR).
2. The structure of the Program, the materials for the Program, the publisher (if any), the English language version (if any), and any consents.
3. The details for use and transfer, the person to contact to use the program and any R&Di contracts when a Knowledge Transfer Department is involved.

### Details of evidence-based validation

#### Details of program validation

##### Basis of the program: Theoretical model and supporting empirical evidence

Integral to an evaluation of an Intervention Program is an explicit statement of the underlying theoretical model and the supporting empirical evidence published in recognized international scientific publications. Such evidence is generally in the form of published peer-reviewed articles or manuals.

There are numerous models in educational psychology that have been confirmed by evidence. Programs can be analyzed by issue, field of intervention and how up to date they are. As a general proposition, reviews of specific research topics and manuals of research review (de la Fuente and Justicia, 2018) describe developments in different subject areas and in relation to different theoretical models.

##### Validation method

Published peer-reviewed reports of research and impact assessments that set out the effects of the Program and the general procedure for each study in sufficient detail for it to be replicable are also required for full evaluation of a Program. To that end, information is required as follows:

###### Study participants

Sample: inclusion and exclusion criteria, type of sample.

###### Instruments

Validity and reliability of instruments used to validate a program, whether part of the program itself or other instruments recognized by the scientific community.

###### Procedure

The details of the procedure should be sufficiently clear to enable replication in new samples. It is essential to provide Ethics Committee approvals and where applicable the Pre-Clinical Registration of the validation study.

###### Data design and analysis

The study design (Anguera et al., 1995; Ato et al., 2013) should be stated in sufficient detail and the data analysis should be consistent with the type of validation intended.

##### Results

The results should be presented clearly and unambiguously relative to the expected effects of Program implementation. This section, depending on the nature of the results submitted and the design, is key to determining the nature of the evidence provided and its strength.

##### References to publications that provide evidence

The list of published research should be provided. That will enable the sufficiency and adequacy of the evidence to be determined.

##### Final evaluation

Following Slavin (2019), there are different levels of evidence:

1. Strong evidence: Results are consistent and designs are robust (experimental or quasi-experimental) and well-implemented.
2. Medium evidence: Results are consistent but the design has a medium level of consistency (correlational designs).
3. Weak evidence: Results have consistency but the design is very weak with descriptive data.
4. Evidence non-existent: No results are provided that constitute evidence and the design is very weak.

## Proposed protocol for the registration of evidence-based educational psychology intervention programs

### Professional context

1. Professional field
  1.1. Optimization of processes of development, teaching, and learning (de la Fuente, 2020).
  1.2. Management of special educational needs and learning difficulties.
  1.3. Academic and career guidance.
  1.4. Research, Technological Development, and Innovation in Psychopedagogy.
2. Specific field
  2.1. Optimization of processes of learning.
  2.2. Optimization of processes of development.
  2.3. Optimization of processes of teaching.
  2.4. Management of special educational needs in development processes.
  2.5. Management of special educational needs in learning processes.
  2.6. Academic guidance.
  2.7. Career guidance.
  2.8. Research, Technological Development and Innovation in Psychopedagogy.
3. Subprogram
  3.1. Psychomotor Development
  3.2. Personal (Emotional) Development
  3.3. Social-Ethical Development
  3.4. Cognitive Development
  3.5. Linguistic Development.
4. Type of program
  4.1. Preventive (primary prevention)
  4.2. Palliative (secondary prevention)
  4.3. Treatment (tertiary prevention)
  4.4 Retrospective (Stage 4 prevention)

### Program details

1. Name of the Program:
2. Problem addressed:
3. Year of creation:
4. Authors:
5. Intellectual Property Registration:
6. Structure OF Intervention Program:
7. Materials:
8. Publisher (if any):
9. Previous English-language version (if any) and associated consents (if any):
10. Translation into English (if any):
11. Details of use and transfer:
12. Contact:
13. Existence of R&D contract for use (if any):

### Details of evidence-based validation

1. Theoretical Basis. Theoretical models on which program is based. Recent evidence:
2. Validation method:
  2.1. Participants.
  2.2. Instruments.
  2.3. Procedure.
  2.4. Data design and analysis.
3. Results obtained: synthesis of results.
4. References to significant publications (peer review system):
5. Evaluation proposal (Slavin, 2019):
  5.1. Strong evidence:
  5.2. Medium evidence:
  5.3. Weak evidence:
  5.4. Evidence non-existent:

## Exemplar completed protocol for the registration of evidence-based based Educational Psychology intervention program: The ALADO program (2012)

### Professional context

1. General area
  1.1. Optimization of processes of development, teaching, and learning (de la Fuente, 2020).
  1.2. Management of special educational needs and learning difficulties.
  1.3. Academic and career guidance.
  1.4. Research, Technological Development, and Innovation in Psychopedagogy.
2. Specific area
  2.1. Optimization of processes of learning.
  2.2. Optimization of processes of development.
  2.3. Optimization of processes of teaching.
  2.4. Management of special educational needs in processes development processes.
  2.5. Management of special educational needs in learning processes.
  2.6. Academic guidance.
  2.7. Career guidance.
  2.8. Research, Technological Development, and Innovation in Educational Psychopedagogy.
3. Subprogram
  3.1. Psychomotor Development.
  3.2. Personal (Emotional) Development.
  3.3. Social-Ethical Development.
  3.4. Cognitive Development.
  3.5. Linguistic Development.
  3.6. Language Learning.
  3.7. Mathematical Learning.
  3.8. Social Learning.
  3.9. Personal Learning.
4. Type of program
  4.1. Preventive (primary prevention).
  4.2. Palliative (secondary prevention).
  4.3. Treatment (tertiary prevention).
  4.4. Retrospective (Stage 4 prevention).

### Program details

Name of the program: ALADO Program to Prevent Alcohol Abuse in Adolescents.

Problem: Prevention of Alcohol Consumption by Adolescents.

Year of Creation: 2012.

Authors: Lead researchers:

- Dr. Jesús Enrique de la Fuente Arias
- Dr. Inmaculada Cubero Talavera

Participating researchers:

- Dr. Francisco Javier Peralta Sánchez
- Dr. María Dolores Sánchez Roda

Intellectual Property:

- Teacher guide: ISBN: 978-84-938657-3-3
- Intervention guide: ISBN: 978-84-938657-2-6

### Structure of the program

ALADO is an online program for the prevention of the consumption and abuse of alcohol. It is aimed at young people aged 12–16 and is split into nine modules that progressively educate participants about the undesirable effects of alcohol consumption. The program provides the essential tools to enable young people to reduce or avoid the consumption of alcohol through management of their own behavior.

#### Objectives of the program

- To make participants aware of the effects of alcohol consumption for adolescents, particularly, on their brains. The program seeks to decisively change attitudes to alcohol and to give participants strategies to regulate their own behavior so that they can avoid excessive alcohol consumption. We offer participants mechanisms to access pertinent information through a fun, attractive online platform, which has been designed and structured to appeal to young people.
- To teach young people that alcohol is a drug that has negative effects on our health.
- To encourage abstinence from alcohol.
- To teach and offer behavioral strategies to truly say NO to alcohol, particularly in social situations where there is peer pressure.

#### How it works

The program is conceived as a school activity to be carried out in the classroom and includes help and guidance for tutors and teachers. However, ALADO is available online and so can also be used in the home. It has been developed to be free-standing so that anyone who wants to can register and use the program content. That gives control to the family and allows the activities to be completed in the home.

#### Can parents participate?

ALADO has a parent-teacher space called Parent-Teacher School with materials that explain and train about the effects of alcohol consumption and the different ways that the problem is addressed at national and regional level. It also offers links to websites and internet portals concerning the prevention of alcohol consumption. Parents and teachers can find valuable reading material for active involvement in the education of their children in a culture based on saying NO to alcohol. By way of example from that material:

- The Guide to Prevention from the National Drug Addiction Plan.
- Alcohol: Clinical Committee reports.
- NIDA prevention program.
- It is always your choice.
- Guidance for SPAN parents and strategies for taking action.

#### Origins of ALADO

Adolescence is a developmental stage of biological and cognitive maturation, which is characterized by experimental, risky behaviors. That prototypical behavior pattern is perfectly reflected in the unfortunately notorious pattern of heavy weekend binge drinking in which abstinence during the week is followed by abuse at the weekend. Although an adolescent might not always see that pattern of alcohol consumption as risky to their health, science has shown that a habit of intermittent intake in a consumption-relapse pattern is associated with neurobehavioral changes, with a tendency toward increased alcohol consumption and an increased risk of addiction in adulthood. The most recent research has shown that young people are unfortunately less susceptible to alcohol intoxication. In other words, adolescents are more resistant to alcohol’s sedative effect and so may drink larger volumes in less time…“an adolescent can take more…” Paradoxically and despite their apparent non-susceptibility to its sedative effects, alcohol affects the adolescent brain more severely than the adult brain, because young brains are still developing.

#### ALADO at the University of Almería

Concerned at the alarming rise in alcohol abuse in our young people, a group of experts working in neurobiological research and educational psychology at the University of Almería joined forces and knowledge on ALADO, a project to develop an ALcohol prevention program for Adolescents (“with wings” in Spanish).

Financed by the Ministry of Innovation, Science and Business of the Regional Government of Andalusia, the aim of the program is to bring about change in capacity and attitudes concerning excessive alcohol consumption in adolescents. ALADO is a pilot that seeks to transfer knowledge derived from research to society, to a group as highly vulnerable as adolescents.

#### Information and education online

This ambitious project aims to develop the first completely free-standing software for secondary school students, parents and teachers to prevent the consumption and abuse of alcohol. The strategy is to give students the necessary information and tools to be able to reflect on the effects of alcohol consumption and to prompt a change in attitudes toward alcohol.

As a pilot project, the expert group in Almería implemented the program in secondary schools to work with students in the 12–16 age range before they had become habituated to the consumption of alcohol. ALADO was implemented in the classroom in curriculum time over a period of 1 year.

Using appropriate evaluation tools, the effectiveness of ALADO was assessed using a pre-post test of the attitudes and behavior of our adolescents before and after their experience of ALADO.

### Materials

- www.alado.es (https://www.miguelturra.es/programa-informatico-pretende-reducir-ingesta-alcohol-adolescentes-wwwaladoes)
- Printed material: de la Fuente et al. (2012).
- Publisher: Publication associated with the Electronic Journal of Research in Educational Psychology. http://www.investigacion-psicopedagogica.org/revista/new/index.php. Education & Psychology R&DI (Spin-Off. University of Almería, Spain).
- Previous English language version: No.
- Translated into English: No.
- Details of use and transfer: Contact: jfuente@ual.es; icubero@ual.es; fjperalta@ual.es; R&D Use Agreement: From OTRI at the University of Almería.

## Details of evidence-based validation

### Theoretical basis: Theoretical model

Model of Competence to interact with alcohol (de la Fuente et al., 2017). The program consists of a total of 24 lessons that facilitate computer-enabled conceptual, procedural and attitudinal learning.^1^

### Validation method

#### Participants

A total of 148 adolescents aged 12–16 took part in three Spanish schools with different social and cultural contexts.

#### Instruments

- *The Scale for the Evaluation of Facts, Concepts, and Principles concerning Alcohol* (EHCP, in Spanish; Cubero and Sánchez, unpublished).^2^ The scale is made up of 38 items related to the effects of alcohol consumption; psychometric analysis of the scale shows reliability (α = 0.827) and consistent construct validity, with three factors: knowledge of facts, concepts, and principles concerning alcohol.
- *The SRQ Self-Regulation Questionnaire* in its 21-item abridged form for adolescents (Neal and Carey, 2005) was used. Spanish version of the abridged SRQ, SRQ-21 (Pichardo et al., 2018). Its reliability (α = 0.826) and validity figures are consistent, with two dimensions: planning and control of actions.
- *The Scale for Assessment of Attitudes to Alcohol* (AAA; Cubero and Sánchez, 2018, unpublished). A total of eight items to evaluate attitudes and values toward alcohol (α = 0.825).
- *The Adjustment Scale in interaction with alcohol*, which contains four times (α = 0.915). This scale belongs to the Inventory of Knowledge, Attitudes, and Interaction with Alcohol (Cubero and Sánchez, unpublished).

#### Procedure

The data-gathering instruments were used during the course of the 2009–2010 academic year as part of the Alado Excellence Project (2007–2010) through an online tool created for the purpose.^3^ Although the data are old, we consider that given their nature they are still highly relevant to the analysis of the variables assessed.

Consent to cooperation by participating schools and pupils was requested from each School Council, the teachers involved and the families (parents) of participating students. The project was approved by the Bioethical Committee of the University of Almería and by the School Council of each participating school. The students participated voluntarily. Parents were informed in writing. Since the participants in the project were still minors, both the parents and the management of the schools gave written informed consent to the research. Data were protected in a registered archive as required by the Spanish Data Protection Act.

#### Data design and analysis

We used a quasi-experimental method with repeat measurement and inferential analysis (ANOVA and MANOVA).

### Results

The results showed a principal effect for the variable Treatment, the variable Level of Self-Regulation and an interaction effect for Treatment × Self-Regulation in the conceptual and attitudinal subcompetency for interaction with alcohol. The results are discussed relative to new technology that allows evaluation and intervention in the prevention of alcohol consumption in adolescents. An important implication of this work is the importance of the psychological variable self-regulation. It also supports the suitability of educational psychology interventions in new technology formats in the prevention of alcohol consumption in adolescents as a commercial activity.

References for publications in the evidence report: de la Fuente et al. (2017, 2019) and Marcos-Pérez (2012).

Final evaluation proposal (Slavin, 2019):

Strong evidence:

Medium evidence:

Weak evidence:

Evidence non-existent:

## Conclusion and discussion: The future of programs in Educational Psychology

In summary, it seems reasonable following Slavin (2019) to propose the adoption of a *Protocol for the Registration of Evidence-Based Educational Psychology Programs*. The proposal made here is preliminary and could be adapted to other areas of Psychology.

It would also be necessary to create a *National Register of Evidence-Based Educational Psychology Programs,* similar to the register that exists for *Psychological Tests* (Commission for Tests Edited in Spain, 2012). Some existing models in the field of education, e.g., Best-Evidence Encyclopedia; BEE; http://www.bestevidence.org and ESSA, https://www.evidenceforessa.org could provide guidance. The Register should be based on anonymous peer assessment by researchers and practitioners with accredited experience in the scientific review of Psychological Programs. It is evident that such assessment would give added value to existing Intervention Programs and allow published programs that have no supporting evidence to be distinguished from those that do have such evidence. It could also act as an endorsement in processes of academic and professional accreditation. It would allow users to distinguish among the many Intervention Programs available (in hard-copy and/or remotely) those that are not evidence-based – and have therefore not been validated – from those that have been. In summary, the proposal made in this article would be a step forward in the scientific improvement of the profession of Psychology in general and Educational Psychology in particular (de la Fuente, 2020).

## Data availability statement

The original contributions presented in the study are included in the article/supplementary material; further inquiries can be directed to the corresponding author.

## Author contributions

All the authors have contributed equally, in the editing and revision of this work. JF and MMV-M made the initial drafting of the protocol. JMM-V and FJP-S made the final corrections and the adaptation of the protocol to the ALADO program. All authors contributed to the article and approved the submitted version.

## Funding

This work was supported by Project I+D University of Navarra (PGC2018-094672-B-I00; Ministry of Science and Education, Spain) and University of Almería (European Social Fund) Project I+D (AL18-SEJ-DO31-A-FEDER).

## Conflict of interest

The authors declare that the research was conducted in the absence of any commercial or financial relationships that could be construed as a potential conflict of interest.

## Publisher’s note

All claims expressed in this article are solely those of the authors and do not necessarily represent those of their affiliated organizations, or those of the publisher, the editors and the reviewers. Any product that may be evaluated in this article, or claim that may be made by its manufacturer, is not guaranteed or endorsed by the publisher.

## Footnotes

^1^ www.alado.es

^2^Cubero, I. and Sánchez, M. C. (2018a). Scale for the Evaluation of Facts, Concepts, and Principles concerning Alcohol (EHCP). Manuscript pending publication Almería: University of Almería.Cubero, I. and Sánchez, M. C. (2018b). Scale for Assessment of Attitudes to Alcohol (AAA). Manuscript pending publication. Almería: University of Almería.

^3^ www.alado.es
